# The Nigral Coup in Parkinson’s Disease by α-Synuclein and Its Associated Rebels

**DOI:** 10.3390/cells10030598

**Published:** 2021-03-09

**Authors:** Jeswinder Sian-Hulsmann, Peter Riederer

**Affiliations:** 1 Department of Medical Physiology, University of Nairobi, P.O. Box 30197, 00100 Nairobi, Kenya; 2 Clinic and Policlinic for Psychiatry, Psychosomatics and Psychotherapy Margarete-Hoeppel-Platz 1, University Hospital Wuerzburg, 97080 Wuerzburg, Germany; peter.riederer@uniwuerzburg.de; 3 Department Psychiatry, University of Southern Denmark Odense, J.B. Winslows Vey 18, 5000 Odense, Denmark

**Keywords:** Parkinson’s disease, substantia nigra, alpha-synuclein, genetics, iron, neuroinflammation, viruses, immunology, aging and cell death

## Abstract

The risk of Parkinson’s disease increases with age. However, the etiology of the illness remains obscure. It appears highly likely that the neurodegenerative processes involve an array of elements that influence each other. In addition, genetic, endogenous, or exogenous toxins need to be considered as viable partners to the cellular degeneration. There is compelling evidence that indicate the key involvement of modified α-synuclein (Lewy bodies) at the very core of the pathogenesis of the disease. The accumulation of misfolded α-synuclein may be a consequence of some genetic defect or/and a failure of the protein clearance system. Importantly, α-synuclein pathology appears to be a common denominator for many cellular deleterious events such as oxidative stress, mitochondrial dysfunction, dopamine synaptic dysregulation, iron dyshomeostasis, and neuroinflammation. These factors probably employ a common apoptotic/or autophagic route in the final stages to execute cell death. The misfolded α-synuclein inclusions skillfully trigger or navigate these processes and thus amplify the dopamine neuron fatalities. Although the process of neuroinflammation may represent a secondary event, nevertheless, it executes a fundamental role in neurodegeneration. Some viral infections produce parkinsonism and exhibit similar characteristic neuropathological changes such as a modest brain dopamine deficit and α-synuclein pathology. Thus, viral infections may heighten the risk of developing PD. Alternatively, α-synuclein pathology may induce a dysfunctional immune system. Thus, sporadic Parkinson’s disease is caused by multifactorial trigger factors and metabolic disturbances, which need to be considered for the development of potential drugs in the disorder.

## 1. Introduction

The neurodegenerative disorder Parkinson’s disease (PD) was first described by James Parkinson in 1817. It is defined by the characteristic and marked poverty in movement. It has been shown to afflict 17 per 100,000 person-years [1]. It is classically associated with the manifestation of resting tremor, bradykinesia, postural instability, akinesia and non-motor symptoms [2]. The motor symptoms are attributed to the deficit of striatal dopamine resulting as a direct consequence of the progressive destruction of the substantia nigra (SN) pars compacta neurons [3].

In incidental Lewy body (LB) disease, there are α-synuclein inclusions with no remarkable neurological symptoms. It exhibits nigral pathology (Braak α-synuclein stage 3–4) marked by SN cell loss (about 46%), and LB and is considered preclinical PD [4]. Thus, α-synuclein aggregates/LB are a formidable contender as an elite member of the multifactorial group of PD etiological factors. The absence of motor symptoms may be due to the operation of some compensatory neuroplasticity mechanisms or the lack of some other factor(s), or a more significant nigral cell loss is required to progress to the symptomatic phase. Axon terminals may be the culprit of triggering the degenerative neuronal process in a retrograde manner, since α-synuclein is involved in the exocytosis of presynaptic vesicles [5,6]. Indeed, early symptoms are evident in around 50–60% of nigro-striatal axon terminal degeneration; in contrast, there is only about 30% loss of neuromelanin (NM) containing dopaminergic neurons in the SN [6,7]. The SN neuronal loss in the early asymptomatic stages of the illness can be attributed to the operation of degenerative processes such as oxidative stress (OS) triggered by misfolded α-synuclein. This notion is supported by the depletion of the cellular antioxidant glutathione (GSH) in the SN in incidental Lewy body disease [8]. However, it appears that the attribute of neuroplasticity wanes or diminishes as the disease progresses. Indeed, adult neurogenesis has been found to be markedly disturbed in post-mortem studies in PD [9], whereas others reported no changes [10]. The loss of these NM containing SN cells accounts for the distinctive “bleached” appearance of this area in the illness. Neuropathological studies reveal a marked SN neuronal loss, reactive gliosis, and the presence of LB in the remaining neurons [11]. The major component of LB pathology is the amasses of misfolded protein, α-synuclein [12]. LB are characteristic of PD, although they are observed also in other neurodegenerative maladies. More importantly, the distribution of LB appears contiguous to the nigral cell depletion, thereby endorsing the importance of these structures to the neuropathogenesis of the disorder.

The cause(s) and the precise mechanism(s) underlying the selective destruction of the dopamine neurons in the SN pars compacta PD remains elusive, despite a surfeit of potential and budding candidates [13], thereby suggestive of a multifactorial involvement in the pathogenesis. Furthermore, these contenders share a common feature; they possess the “license to kill” as demonstrated by their propensity to trigger cytotoxic events resulting ultimately in the characteristic dopamine cell death in PD. In addition, there are two pertinent issues to be considered; firstly, that there is more than one culprit causing the disease and secondly, they may collude and share or employ common destructive pathways: a case of “All roads lead to Rome”. The multifactorial etiology coupled with age-related risk and some underlying genetic predisposition may contribute to the molecular changes that provoke neurodegeneration [14]. Indeed, PD is an age-related disorder, and its prevalence increases with age.

## 2. Typical Features of Parkinson’s Disease

### 2.1. Why Dopaminergic Neurons Die?

The selective vulnerability of nigral dopaminergic neurons to the onslaught of PD compared to other central dopamine pathways may be related to a collection of features including structure, neurophysiological features, and metabolic requirements. The nigro-striatal tract comprises of the major motor pathway originating from the SN pars compacta with dense axonal arborization mainly in the striatum. Thus, loss of the SN dopamine neurons in the disease process has marked consequences such as degeneration of the SN pathway. Furthermore, the SN tract contributes to a significant (around 80%) proportion of the total brain dopamine in contrast to other dopaminergic circuits such as the mesolimbic, meso-cortical, and tuberoinfundibular systems. This ascribes for the profound effect of SN neurodegeneration and striatal dopamine depletion to the interruption of motor feedback circuits and thus motor deficit in PD and explains the powerful action of levodopa therapy. It appears that the axonal arbor size of the neurons corresponds directly to its susceptibility. Thus, the extensive nigral neuronal connectivity probably warrants high demands for proteostasis and mitochondrial energy, thereby increasing the susceptibility to cytotoxic processes such as OS, genetic mutations, and deterioration of proteasomal activity [15,16,17]. The proteasomes are involved in the destruction of ubiquitinated or damaged proteins and this ascribes for an age-related deposition of aberrant proteins in the brain. In addition, advancing age may compromise the physiological compensatory protective cellular mechanism(s), such as mitochondrial oxygenation, chaperone activity in lysosomal autophagy, and proteasome-mediated proteolysis. In turn, this may account for the age-related propensity for the brain to accumulate rogue proteins such as modified α-synuclein.

Interestingly, α-synuclein is implicated in most of the fundamental pathological features observed in PD, including the dysfunction of metabolic and physiological processes and neuroinflammation. Thus, these α-synuclein malfunctions may represent the “rebels” associated with the neurodegeneration occurring in PD. The phenomenon of α-synuclein aggregates in LB is a selective neuropathology finding in PD and other related α-synucleinopathies such as diffuse Lewy body disease (DLB) and multiple system atrophy (MSA) [18]. The aggregates of misfolded α-synuclein are considered to exert a pivotal role and have received notoriety in the pathogenesis of PD, although connectome-mapping studies have shown inconsistent correlation between LB pathology and neuronal cell death [19]. Nevertheless, α-synucleinopathies are multidimensional, and it is very likely that other factors come into play in the development of the diseases.

Therefore, for advocating effective therapeutic strategies and perhaps halting the progression of the disease, it is fundamental to elucidate the underlying cellular and molecular mechanism(s) that orchestrate α-synuclein fibrillization, accumulation, and subsequent LB formation. α-Synuclein appears to exert a physiological role in the mediation of neurotransmitter release from synaptic vesicles, although α-synuclein depletion does not produce any marked effect on neuro-synaptic transmission [20]. It appears that it operates as a “double agent”, and in the misfolded form, it adopts a sinister and deadly role. Indeed, the pathological form of α-synuclein is structurally and physiologically different from the native form [21]. In addition, there are different “strains” of α-synuclein produced intracellularly, depending on the internal environment and misfolded α-synuclein seeds. Furthermore, the more condensed α-synuclein aggregates correspond to a more aggressive form. This concept is supported by the highly dense α-synuclein that appears as glial cytoplasmic inclusion in multiple systems atrophies, which is about 1000-fold more lethal compared to the LB aggregates in the PD neurons. This probably attributes to the rapid rate of progression of degeneration in multiple systems atrophy in contrast to that in PD.

Thus, these aggregates have the potential to summon lethal militia that can evoke cellular devastation. Indeed, α-synuclein pathology is subversively associated with collateral cellular carnage primarily advocated by producing malfunctions in the mitochondria, synaptic organization, neurotransmission, plasticity, and cytotoxic processes such as oxidative stress [22]. It has been suggested that during the formation of mature LB, there are interactions between newly formed α-synuclein aggregates and mitochondria, resulting in the sequestration of proteins and mitochondrial organelles [23]. This summons a severe breakdown in the mitochondrial activity.

Therefore, to block or hinder α-synuclein neuropathology would result in direct consequence of slowing/eventually halting the disease process underlying PD. So, it is imperative to understand the conditions, mediators, and mechanisms that are responsible for its manifestation to ascertain the effect of LB pathology on neuronal survival.

### 2.2. Lewy Body Pathology

LBs are the hallmark of PD. Their occurrence especially in the degenerating dopaminergic neurons to the substantia nigra pars compacta has been suggested to be evidence for their critical role to destroy these neurons. However, there is still no proof as to their role in the pathology of PD [24,25]. In fact, LB formation may be a consequence resulting from the dysfunction of several cellular mechanisms, including aberrant misfolded proteins, ubiquitin–proteasome system dysfunction, oxidative and nitrosative stress, mitochondriopathy, dysfunction of metal homeostasis, synaptic disturbances, and dysfunction of axonal and dendritic transport, all of which play a major role in the accumulation of respective cellular compounds in LB [26,27,28,29,30,31]. Indeed, so-called non-α-synucleinopathies such as iron storage disease, mitochondrial dysfunction, and lysosomal disorders are particularly susceptible to LB formation [32].

In particular, α-synuclein has been identified as a major component of LB [12,33] and Parkin has been found to be a part of LB disease [34]. Indeed, the enrichment of insoluble forms of α-synuclein is an indication for being a key component of LB [35,36]. More importantly, the formation of α-synuclein oligomers may exert cellular toxicity via irregular mitochondrial function, membrane destruction, damage to the protein clearance system, and amplifying OS [37].

In addition, the accumulation of advanced glycation products (AGE) on nucleotides, lipids, and peptides/proteins are important components on the aging process [38] as they are structural crosslinkers that cause the transformation of soluble neurofilament proteins to insoluble LB [39,40].

**Figure 1:**
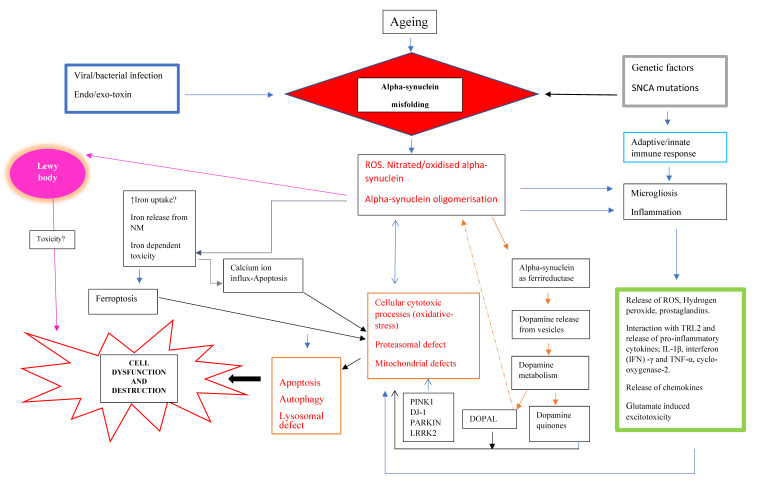
α-synuclein associated neurodegenerative pathways in Parkinson’s disease (PD).

All this demonstrates that the LB formation involves a complex interaction of α-synuclein fibrillization with a number of different compounds from various dysregulated cellular mechanisms. Despite the fact that LB finally destroy neurons, it remains open as to whether LB formation is of primary/early importance in the pathology of dopaminergic cell death in PD. Indeed, the composition of core and halo of LB points to the dysfunction of basic cellular processes in early stages of the pathology with the consequence of late LB formation (Figure 1).
DOPAL3,4 dihyroxyphenylacetaldehydeNMNeuromelaninROSReactive oxygen speciesTRL-2Toll-like receptor 2TNF-αTumor necrosis factorSNCASynuclein AlphaPINK 1PTEN-induced kinase 1DJ-1Protein deglycasePARKIN(PARK2), an E3 ubiquitin ligaseLRRK2Leucine-rich repeat kinase 2

The accumulation of α-synuclein may be induced by particular factors or molecules coupled with an underlying genetic component. Subsequently, nitrate/oxidative stress-induced modification may favor its oligomerization and aggregation. These aggregates have the propensity for eliciting pathways that lead to the neuronal dysfunction and finally cell death. These include neuroinflammation, proteasomal defect, iron-dependent ferroptosis, autophagy, and apoptosis. In addition, α-synuclein can operate as ferrireductase and cause the release and subsequent breakdown of dopamine to neurotoxic metabolites such as, DOPAL.

### 2.3. Disturbed Metabolic and Physiological Mechanisms

As mentioned, α-synuclein may disrupt metabolic processes such as mitochondrial function. Additionally, it may be closely related to iron dyshomeostasis and dopamine regulation. Consequently, this may initiate cellular destructive mechanisms. In addition, the α-synuclein containing LB may perhaps not directly produce neurodegeneration. Instead, it is the cellular detrimental effects produced by the intermediate species during the maturation of LB. The production of LB may be ascribed at least in part [36] to the deficiency in SN complex I in PD [41,42,43]. Additionally, other cytotoxic processes such as OS may be closely associated to the reduction in nigral complex I in PD. A plethora of evidence supports OS as a major contributor of the progressive SN neuronal degeneration via reactive oxygen species (ROS) or free radicals-mediated apoptotic cell death [17,44]. Guzman and colleagues [45] showed that blocking calcium-dependent voltage-L type channels (Cav1) attenuated the vulnerability of nigral dopamine neurons to OS-related mitochondrial dysfunction and autophagic stress, thereby supporting the involvement of calcium ion-driven apoptotic mechanism(s) in cell death. This notion is endorsed by selective molecular and cellular changes in the SN in PD, including a reduction in cellular antioxidant glutathione (GSH) [8,46,47], elevation of total iron and iron (III) [48], a reduction in ferritin [49], increased nicotinamide adenine dinucleotide phosphate oxidases [50], and raised lipid peroxidation [51]. A lipid peroxidation product, 4-hydroxynonenal, is highly reactive and can induce calcium-mediated apoptotic cell destruction and proteasomal dysfunction [52]. So, 4-hydroxynonenal may impair the proteasomes involved in the degradation of α-synuclein, thus funding its accumulation (Figure 1).

Additionally, elevated iron induced build-up of lipid peroxides and decreased glutathione content in the SN may prompt ferroptosis [53]. Ferroptosis is an iron-dependent programmed cellular destruction pathway, which is distinct from apoptosis. This notion supports the advocation of iron chelators, which are inhibitors of lipid peroxidation and lipophilic antioxidants as potential targets for cellular protective therapy in PD and other disorders exhibiting iron accumulation [54]. The nigral iron deposition in PD can augment the misfolding of α-synuclein and thereby enhance its aggregation and cellular detrimental effects, similar to the iron-related hindrance in the formation of well-ordered amyloid beta peptide aggregates [55]. Furthermore, oligomeric structures produced from α-synuclein inclusions have been shown to be structurally related to amyloid beta peptide fibrils found in Alzheimer’s disease [56]. This may indicate some common iron-mediated neurodegenerative pathway [57,58,59]. In addition, an oxidative environment supports the aggregation of α-synuclein, thereby exacerbating the neurodegenerative pathology load [60,61]. Conversely, it has been demonstrated that elevated levels of α-synuclein oligomers can activate both apoptosis via calcium ion influx and ferroptosis by iron-dependent ROS production and lipid peroxidation [62].

Another fascinating finding related to α-synuclein is that it can operate as cellular ferrireductase [63]. It reduces ferric iron (III) to ferrous iron (II) using copper as a cofactor and NADH (Nicotinamide adenine dinucleotide reduced form) as an electron donor. This revelation has a number of important implications for α-synuclein pathology in the cascade of neurodegeneration [60]. Firstly, since ferrireductase utilizes NADH, it may disturb the mitochondrial NADH plus hydrogen ion redox equilibrium at complex I in the electron transport chain, thereby resulting in its documented decrease in SN in PD. Additionally, ferrireductase may contribute to iron dyshomeostasis in the SN, as reported in PD. Alternatively, even if it is not linked to the iron deposition, nevertheless, ferrireductase can reduce the excess iron to the toxic ferrous iron (II) form. Consequently, iron (II) ions can react with hydrogen peroxide in the Fenton reaction to generate ROS such as hydroxyl radicals. Subsequently, these ROS can produce cellular destructive effects through the oxidative modification of proteins, lipids, and DNA. In addition, the overproduction of ROS can tip the balance between pro-oxidation and anti-oxidation, resulting in oxidative stress. Eventually, this may overwhelm cellular defense antioxidants such as glutathione and account for its depletion in the SN in incidental Lewy body disease and PD [17].

There is some evidence to suggest that α-synuclein or ferrireductase can influence dopamine synthesis [5] trafficking, and metabolism [64]. In addition, α-synuclein aggregates may damage dopamine vesicles, resulting in its release into the cytoplasm and consequent breakdown (Figure 1). Dopamine can be oxidized non-enzymatically to produce reactive molecules, such as dopamine quinones. In addition, the enzymatic (monoamine oxidase) oxidation of dopamine results in the formation of 3,4 dihydroxyphenylacetaldehyde (DOPAL). DOPAL is neurotoxic and supports the oligomerization and construction of α-synuclein deposits [65]. This metabolite supports the aggregation of disordered α-synuclein. Interestingly, DOPAL was found to be more effective than dopamine at oligomerizing α-synuclein and the formation of quinone adducts with it [66]. Ferrireductase enhances cellular sensitivity to DOPAL neurotoxic effects, thereby exacerbating the dopaminergic neuron causalities. Thus, the advocation of the gold-standard PD treatment, L-dopa/carbidopa or benserazide, may intensify dopamine cell loss, although there is not much evidence for this assumption. Nevertheless, the dopamine metabolites and ROS may induce the nitration of α-synuclein, which prompts its propensity to misfold and aggregate.

### 2.4. Immunological Aspects

The release of oxidized/nitrated α-synuclein present in LB from dying/dead dopamine neurons in the SN may recruit innate and adaptive immune responses. Indeed, the occurrence of inflammation is supported by the reactive microgliosis reported in the SN in PD [67]. Microgliosis represents the occurrence of an immune defense reaction, since microglia are the principle immune cells in the brain. It is an important contender in inflammation that is associated to neurodegenerative processes in PD (Figure 1).

It has been suggested that nitrated/modified α-synuclein may be principal in microglia activation [68]. These reactive microglia cells interact with Toll-like receptor 2 (TLR2) to release inflammatory mediators such as the potent pro-inflammatory cytokine interleukin 1 beta (IL-1β). In vitro studies using rat primary cultures attest that α-synuclein releases matrix metalloproteinases (MMP-1, -3, -8, and -9), which in turn activate microglia and activate protease-activated receptor-1. The protease-activated receptor-1 further intensifies microglial inflammation [68]. The inflammatory species released from the microgliosis may generate ROS that provoke cytotoxic processes, thereby creating a hostile environment for the surviving dopamine neurons [69]. Interestingly, iron also triggers the activation of microglia and the release of an array of ROS, hydrogen peroxide, prostaglandin E2, interleukin 1 beta (IL-1β), tumor necrosis factor (TNF)-α [70]. Thus, the misfolded α-synuclein in LB serves as an internal activator for inflammatory reactions in PD, which may contribute richly to the neuron massacre.

Microglia are major histocompatibility complex (MHC) class II expressing cells, and they are inclined to bind to aggregated proteins (such as α-synuclein), which are then presented to a cluster of differentiation 4 (CD4+) T cells, thereby exercising a protective role. Interestingly, Arlehamn and colleagues [71] found profound α-synuclein-specific T cells reactivity close to the time of motor symptoms onset; however, this weakened as the disease progressed. A dysfunctional peripheral immunity is reflected by the reduced CD4+T cells. The diminishing α-synuclein reactive T cells correspond with a switch of role from cellular protective to a pathogenic one. It is highly likely that inflammation is likely to exert both a protective and pathogenic role. In the early stages of the disease, it probably executes a defensive approach against the modified α-synuclein structures; however, as the disease progresses, it adopts a more ominous function via the production of pro-inflammatory cytokines and ROS. Alternatively, there may be another trigger agent such as some endo/exotoxin that activates the microglia and associated pro-inflammatory molecules, which is secondary to the early inflammation evoked by the modified α-synuclein aggregates released from the dying dopamine neurons in the SN. Thus, the cellular destructive role of inflammation may represent an epiphenomenon rather than a causal factor; nevertheless, it can exacerbate degeneration. This idea is supported by the microgliosis-related release of pro-inflammatory mediators, including cytotoxic cytokines, which can competently orchestrate neuronal damage. Studies using PD post mortem brain tissue show an elevation in the cytokines, IL-1β, IL-6, interferon gamma (IFN-γ), TNF-α [72,73,74,75] and cyclooxygenase-2 (COX-2) [76]. Although the precise mechanisms of COX-2-mediated dopaminergic cytotoxicity remain controversial, nevertheless, its ability to evoke neuronal destruction via inflammatory and oxidative processes is well established. Similarly, an elevation in cytokines was found in SN and striatum in animals treated with dopamine neurotoxins such as 1-methyl-4-phenyl-1,2,3,6-tetrahydropyridine (MPTP) and 6-hydroxydopamine [77]. Collectively, these observations endorse a definitive association between pro-inflammatory cytokines and α-synuclein aggregate related neuron degradation.

## 3. Risk Factors for PD

### 3.1. Aging

Advancing age is a major risk factor for the development of PD [78]. Epidemiological studies [79] suggest that the prevalence of PD increases by a factor of approximately 10 between 50 and 80 years of age. Interestingly, an age-related increase in NM and iron in the brain has been reported [80], thereby increasing the risk of neuronal destruction with age. NM exerts a malevolent role. Indeed, it can offer cellular protection by chelating iron and thereby blocking iron-mediated ROS (reactive oxygen species) production and cytotoxic processes such as OS [58,81]. However, this process might be limited by the iron-binding capacity of NM, and as toxic compounds accumulate in the cytosol, a release of iron from NM has been suggested. As such, iron can evoke degeneration via microgliosis. Therefore, the increase of NM and iron with age can be regarded as a profound risk factor for triggering PD. NM has been shown to be an immune stimulator in vitro [82]; if this attribute exists in vivo, then it could be postulated that it may trigger the inflammatory cytotoxic cascade in the SN neurons in early PD. In addition, NM containing organelles exhibit a high expression of MHC Class I in dopaminergic neurons, and this may trigger toxic inflammatory processes.

Nevertheless, it seems implausible that age-related decline is the sole contributor to the degenerative process, perhaps it is a prerequisite for the development of PD or for that matter any other neurodegenerative disorder. It is highly likely that there must be some discriminating attribute(s), which define(s) the precise configuration required to determine characteristic pathology manifested in different neurodegenerative disorders. Furthermore, the age-related association to PD is not supported by the difference in the anatomical distribution of cell loss, as in PD, it appears on the ventral tier of the SN in contrast to the age-related dorsal tier. In addition, “normal” aging ascribes for SN neuronal loss at a rate of about 7.4% per decade, in contrast to the marked loss (approximately 60%) in clinical presentation of PD [83]. This suggests the operation of selective degenerative mechanisms coupled with aging.

### 3.2. Genetic and α-Synuclein-Related Pathological Processes in PD

In 1997, two discoveries were decisive to a more causal understanding of the pathology of PD and other neurodegenerative disorders. First, Polymeropoulos et al. [84] discovered a missense mutation in the α-synuclein gene in familiar PD, and second, Spillantini et al. [12,33] identified α-synuclein as a major component of LB and Lewy neuritis. These discoveries were and still are seminal for both hereditary forms of PD and the concept that toxic properties of mutated proteins underlie sporadic PD, which is the much more common type of PD. It has been suggested that approximately 10% of the PD cases are of the familial type with mutations including Parkin, PINK1, DJ-1, LRRK-2, and SNCA (α-synuclein) genes, and the remaining bulk of the PD are of the idiopathic type (Figure 1).

The elevated aggregates may be the result of an over production coupled with factors that are conducive to the conformational change of α-synuclein. Indeed, elevated α-synuclein levels may induce aggregation mechanisms that are nucleation-dependent. So, after a critical level of α-synuclein is attained, intense aggregation ensues. The driving force behind the α-synuclein aggregation in familial PD is the genetic mutations of this protein. This begs the question of the causal facilitator for α-synuclein amassing in the case of the idiopathic form of the disorder. Perhaps there is some key failing in intracellular α-synuclein homeostasis. There are a number of factors that can contribute to the accumulation of α-synuclein such as a reduction in its degradation or clearance of rapid prion-like cellular spread or/and an excess production of the protein. Furthermore, the rate of spread of α-synuclein pathology may directly correlate with progression of the illness, although this notion still needs to be verified [85].

Following the accumulation of α-synuclein in the neurons, it spreads and propagates from infected cells to healthy ones in a characteristic prion-like mode [86]. This notion is supported by the presence of LB in grafted fetal dopaminergic neurons in PD patients [87]. There are a number of methods postulated for the uptake of pathologic α-synuclein by healthy cells including diffusion or endocytosis [88]. The mechanism of uptake appears to be dependent on the species of α-synuclein. For instance, fibrils and oligomeric α-synuclein favor endocytosis for cellular internalization [68]. In contrast, the monomeric form of α-synuclein is transported by diffusion [68]. At present, it is unclear whether the various mechanisms for internalization may lead to a different localization of α-synuclein within the cell, which may in turn affect its aggregation in the recipient cell. Alternatively, tunneling nanotubes can be used for the direct transfer of misfolded α-synuclein within the neuron cells [89]. This allows for the rapid and effective propagation of α-synuclein as demonstrated in primary human brain pericytes obtained from post-mortem PD brains [90]. Interestingly, the LRRK2 gene has been implicated in both the aggregation and propagation of α-synuclein [91]. Furthermore, studies using PD-derived induced neuronal stem cells show that the loss of LRRK2 decreased the aggregation of the rogue protein [92]. This endorses the assertion that mutation of the gene LRRK2 is a risk factor for both the sporadic and familial form of PD [91].

There is a genetic involvement in the misfolding of α-synuclein in the pathogenesis of the familial form of the disease. Indeed, the α-synuclein gene polymorphisms may confer a predilection for its accumulation and thus pose a threat to the development of PD [93]. The mutations observed include several point mutations of α-synuclein in the center region of its helix and the deletion at its C terminus, which confers in its predilection to aggregate and exert neurotoxic effects. Interestingly, for this form of PD, there are specific mutated α-synuclein genes that determine the age of onset, whereby mutations such as A30P, A53T, E46K, and G51D are associated with the early onset, and H50Q is linked with the late onset of the disease [94]. Possibly, the early onset genes are more aggressive and produce abnormal α-synuclein pathology at a more rapid rate, thereby precipitating the disease at a younger age. Under physiological conditions, the ubiquitination of α-synuclein by ubiquitin ligase (SIAH-2, Nedd4) promotes its degradation by proteasomes and lysosome-chaperone mediated autophagy [95]. Lysosomes are the major degradative mechanisms in case of aggregates and oligomers of misfolded α-synuclein. Perhaps in the disease state, the protein clearance mechanisms are defective or may be overwhelmed, resulting in the accumulation of the oligomerized α-synuclein. It is fascinating that α-synuclein mutations A30P and A53T blight the chaperone-mediated autophagy by binding to the lysosome-associated membrane protein type 2a receptor [96]. A similar state may be operative in idiopathic PD; thus, the amassment of oligomers is related to a faulty lysosomal protein degradative pathway, and they are subsequently released from the dying cells and initiate or support the propagation of α-synuclein/LB pathology.

The most critical region seems to be the region from 32 to 58 of N-terminal lipid binding alpha helix domain of α-synuclein [97]. Of particular interest are recent studies to enlighten the structure of native and pathological forms of α-synuclein protein. Such structural information, including techniques such as solid-state nuclear magnetic resonance (NMR) and cryogenic electron microscopy, may be suitable to distinguish pathological phenotypes including PD, DLB, and MSA [98].

α-synuclein mRNA has a structured iron-responsive element (IRE) in its 5′ untranslated region (5′ UTR) that controls translation [99]. At low concentrations of iron, IRE is bound by iron regulatory protein (IRP). However, at high concentrations, IRP is bound by iron, which causes mRNA to undergo translation [99]. Indeed, the concentration of iron has been shown by using different analytical methodologies to be significantly increased in the SN of PD [48,59]. Some of the factors responsible for changes of α-synuclein may include a neurotoxin, amphipathic molecules (such as pesticides, herbicides), the presence of metal ions, a reduction in pH, and an increase in α-synuclein concentration. These factors/conditions may cause the modification of α-synuclein by its nitration or oxidation, which is a process that is mediated by post-translation. This modification occurs early in the process of α-synuclein aggregation and is likely to be responsible for its accumulation [30,31].

**LRRK2** gene mutations cause monogenic types of PD. From a clinical point of view, this type of PD is quite similar to idiopathic PD [100]. LRRK2 gene mutations increase its activity. By this, several cellular pathways are involved in its pathological feature, such as disturbances of protein synthesis, mitochondrial activity, autophagy, and microtubule function in neurons and immune cells [101].

It has also been shown that LRRK2 is associated with α-synuclein pathology [102,103]. The PD-linked G2019S mutation in LRRK2 enhances α-synuclein propagation efficiency by Ras-related protein Rab-35 phosphorylation [104]. The interaction of LRRK2 and α-synuclein has been reviewed recently in detail by O´Hara et al [105] Indeed, this interaction has been challenged, as post-mortem brain analyses have not shown LB in LRRK2-induced PD [105,106]. Experimental studies demonstrate that the expression of familial mutant G2019S LRRK2 does not dramatically elevate α-synuclein or neurodegeneration in neurons [107]. In addition, experiments show that LRRK2 inhibitors did not reverse motor phenotypes, pathological α-synuclein accumulation, or neuron loss, indicating that LRRK2 is not necessary for α-synuclein pathogenesis [108]. Therefore, neuroprotective clinical trials with LRRK2 inhibitors will be necessary to finally show benefits for patients with LRRK2-induced PD or even sporadic PD [109,110].

In autosomal recessive parkinsonism, the products of mutated genes **PINK1** and Parkin are mitochondrial assassins (Figure 1). PINK1 clusters outside mitochondria recruit Parkin, which in turn ubiquitinates the outer mitochondrial membrane proteins to autophagy [111]. Therefore, it has been [112] and is still plausible to assume that in sporadic PD, there must be toxicity arising from a variety of chemical compounds or even viruses, which cause mutations of proteins, including α-synuclein. Here, mitochondria are of special interest, as a loss of respiratory chain activity has been reported independently by three research groups in 1989 [42,43,52]. In line with this, PINK1 is involved in mitochondrial quality control [113]. As such, it interacts with and removes excess α-synuclein, which prevents mitochondrial deficits and apoptosis [114,115]. Evidence for this protective action of PINK1 are experimental studies showing that PINK1 defects cause mitochondrial and proteasomal dysfunction and α-synuclein aggregation [116,117,118], while an inhibition of mitochondrial fusion by α-synuclein is rescued by PINK1, Parkin, and DJ-1 [119].

**DJ-1** has protein chaperone-like activity and exhibits the properties of a protease, deglycase, and a transcriptional regulator that protects mitochondria from OS in suppressing ROS production [120]. DJ-1 protects neurons against the aggregation of α-synuclein and oligomer-induced neurodegeneration [121,122]. Familiar mutations in DJ-1 cause early-onset PD. Reduced activity of DJ-1 due to mutations or OS has been suggested to lead to an accumulation of glycated α-synuclein and its aggregates [123]. In addition, knocking down endogenous DJ-1 renders cells more susceptible to oxidative damage, while overexpression of DJ-1 inhibited protein aggregation and cytotoxicity caused by A53T human α-synuclein [47], DJ-1 is active only in an oxidizing environment in which it inhibits α-synuclein nucleation and remodels mature α-synuclein fibrils in vitro [124].

**PARK 2** gene encodes parkin, an E3 ubiquitin ligase. It is mutated in about 50% of all autosomal recessive PD cases by a probable loss-of-function phenomenon [34]. Parkin interacts with and ubiquitinates the α-synuclein interacting protein, synphilin-1 [125]. Overexpression of parkin decreases the sensitivity to proteasome inhibitors, while antisense knock-down of parkin increases the sensitivity of proteasome inhibitors [126]. Parkin binds, ubiquinates, and targets depolarized mitochondria for destruction by autophagy. Under mitochondrial stress, parkin does not translocate to mitochondria to induce mitophagy [127]. These data show that there is a convergence of parkin, PINK1, and α-synuclein on mitochondrial dynamics and especially in the mitochondrial stress response [127]. The importance of parkin in the pathology of PD is demonstrated also in the colocalization of α-synuclein and parkin in LB.

While those genes have been conclusively linked to PD, the link to ubiquitin carboxyl-terminal hydrolase L1 (**UCH-L1**) remains controversial. Although the I93M mutation in the UCH-L1 gene causes autosomal dominant PD due to a decreased hydrolase activity with a decrease in the availability of free ubiquitin and impaired clearance of proteins, other data demonstrate a differential role of UCH-L1 function under normal and pathological conditions [128,129,130]. Of interest are experimental studies using the rotenone model, showing that in this PD model, UCH-L1 undergoes nitrosylation, which alters its catalytic activity and induces structural instability with the consequence of a faster aggregation of α-synuclein [131].

### 3.3. Neurotoxins

In addition, neurotoxins such as 1-methyl-4-phenyl-1,2,3,6-tetrahydropyridine (MPTP), paraquat, and rotenone augment α-synuclein accumulation probably by inhibition of the ubiquitin–proteasome clearance system [132] (Figure 1). Interestingly, the accumulation of this protein can serve a protective or malevolent role. In the case of paraquat, the elevation of α-synuclein advocates a neuroprotective part by virtue of enhancing the levels of a chaperone protein (HSP70), whereas it adopts a more sinister function and exacerbates MPTP-related toxicity [133]. MPTP has been advocated to disturb the respiratory chain activity. More recent experimental studies demonstrate that MPTP accelerates the rate of α-synuclein aggregation even in the absence of components of the mitochondrial complex [134,135,136]. This interaction of α-synuclein and the neurotoxin MPTP is validated by studies showing resistance to degeneration of MPTP in α-synuclein null mice [137,138]. Therefore, the notion is of interest that MPTP causes a significant decrease of dopamine, tyrosine hydroxylase, and dopamine metabolites due to a degenerative process of dopaminergic neurons in the SN without the formation of inclusion bodies [137,139,140,141,142,143].

However, other neurotoxins, such as metamphetamine, ubiquitin–proteasome inhibitors, and the continuous administration of rotenone (but not discontinuous administration even at high doses) cause the formation of α-synuclein-containing inclusion [133]. The crucial point seems to be that prolonged inhibition of the mitochondrial respiratory chain may cause a loss of the ubiquitin–proteasomal activity [133]. In addition, lipid alterations in membranous compartments promoted by brain aging and PD-like injury, for instance triggered by MPTP, may have an effect on α-synuclein aggregation [144].

### 3.4. Infectious Agents

A possible association of PD with a viral (influenza A) infection was first suggested after the encephalitis lethargica pandemic (1916–1929), although the precise etiology of encephalitis lethargica remains unclear, and other factors such as environmental toxins/or autoimmunity have been suggested [145]. Nevertheless, a viral infection may lead to the inflammation of brain parenchyma, which is known as encephalitis. The post-encephalitic parkinsonism is primarily due to some common changes related to post infection with an element of autoimmune operation [146,147]. Atypical/post encephalitis mainly caused functional damage to basal ganglia, induction of neuroinflammation, and hypoxic brain injury coupled with parkinsonian-like motor features. In addition, similar to idiopathic PD, it exhibits some motor deficits derived from mid brain dopamine deficiency and thus are responsive to dopamine replacement treatment (levodopa and carbidopa or benserazide). Subsequently, there were reports of parkinsonism related to other viral infection such as H5N1, herpes simplex virus 1 (HSV-1), Japanese encephalitis B, St. Louis viral encephalopathy, coxsackie virus, Western equine encephalitis virus (WEEV), Epstein–Barr and human immunodeficiency virus/HIV [148,149]. Interestingly, post-encephalitic parkinsonism does not exhibit any α-synuclein pathology [150], in contrast to some of the other viruses that manifest parkinsonism. Indeed, H5N1 influenza virus was reported to produce microgliosis and α-synuclein aggregation in mice [151]. In addition, influenza virus A and HSV-1 may precipitate parkinsonian-like features due to inflammation and disruption of cellular processes such as autophagy. Subsequently, a malfunction of autophagy may contribute to the build-up of misfolded α-synuclein depending on the virus involved (Figure 1). Amazing results were documented in mice infected with WEEV; they showed many pathological changes similar to those observed in PD, including loss of dopamine neurons in the SN pars compacta, reactive microgliosis, and phosphoserine129 α-synuclein aggregates in the mid-brain [152].

Human immunodeficiency virus (HIV) positive patients can exhibit an array of motor abnormalities including parkinsonism and marked neuropathology in the brain dopaminergic regions, including the SN. Around 5% of the HIV-infected patients develop parkinsonian-like symptoms. It initiates an inflammatory reaction that subsequently leads to activation of microglia in the basal ganglia, SN α-synuclein expression (in 16% of the HIV positive cases, [153]), dopaminergic dysfunction, and progressively damages the blood–brain barrier, similar to that observed in PD. The HIV protein trans-activator of transcription (Tat) may disturb dopaminergic neuronal function in these patients. HIV infection, particularly that associated with dementia, appears to influence dopamine metabolism in the brain [154,155] which may lead to a reduction of dopamine and ascribe for some of the HIV-related motor parkinsonian deficits. Interestingly, the neurological symptoms are seen in the early stages of the infection. This corresponds with the early dopamine depletion (44%) documented in Simian immunodeficiency virus-infected non-human primates [155]. In HIV dementia, there is a decrease in dopamine transporter (DAT) levels in the caudate–putamen region compared to HIV without dementia and controls [156]. These findings are suggestive of a DAT-associated reduction of dopamine content at the synapse and thus compromised dopaminergic neurotransmission.

Recently, SARS-CoV-2 virus has caused the COVID-19 pandemic, which has ravaged the human population. Although it is a respiratory syndrome, there are reports of neurological effects. These neurological (and other) symptoms may be para-infectious, and they are primarily generated by the acute inflammation observed in the illness. This state of acute inflammation may be due to the direct viral infection; or, it might be due to the body’s innate and adaptive immune response to the infection that may lead to inflammation of the vascular system or the peripheral and central nervous system. In a French study, 84% of the intensive care SARS-CoV-2 infected patients exhibited neurological symptoms and 79% exhibited delirium or acute encephalopathy [157]. In contrast, others reported only 16.5% [158]). The high occurrence of delirium reported by Helms and co-workers was attributed to the acute SARS-CoV-2 infected patients that had been admitted for acute respiratory distress syndrome (ARDS). The inflammation related to ARDS is initiated by hypoxia [159]. Interestingly, Fazzani and colleagues [160] reported antibodies to coronavirus in the cerebrospinal fluid of PD patients, thereby suggesting the involvement of an infectious agent and/or the inflammatory system in the pathogenesis of the illness.

The presence of the SARS-CoV-2 virus in various areas of the brain including the cerebrum, cerebellum, and olfactory bulb endorses its involvement in the neuropathological changes exhibited in the brain [161]. The most common finding is microgliosis (42.9%) similar to PD, thereby reflecting the involvement of inflammatory processes in cell death. A recent study reported that patients infected with SARS-CoV-2 may also exhibit more grave neurological symptoms including encephalitis, demyelinating disease, and acute cerebrovascular incident [162]. These changes may be related to cytopathogenic effects of the virus or the inflammatory “cytokine storm” produced by it. Fortunately, the incidence of these severe neurological changes caused by SARS-Cov-2 has a low occurrence (1.6% for strokes, [161]).

Encephalopathy accounts for the neurological manifestations, as is the case with other viral infections. Hyposmia is a feature that is commonly observed both in SARS-CoV-2 infected patients [163] and PD [164]. In PD, it has been attributed to dopaminergic malfunction in the olfactory bulb. Interestingly, α-synuclein pathology is also present in this area in PD. The route of entry of SARS-CoV-2 to the brain via ACE receptors is through the olfactory bulb, which results in the inflammation of that area, resulting in hyposmia. So, in the case of PD, perhaps there is an inflammatory mediator (virus/neurotoxin) that also gains access to the brain via the unprotected part of the brain, the olfactory system. These findings strongly support the involvement of an exotoxin/infectious agent that is instrumental in the pathogenesis of the disease. Post-mortem studies using SARS-CoV-2 infected brain tissue [165] exhibit neuronal necrosis, which is characteristic in neurodegenerative cell death and is probably related to some inflammatory cytotoxic molecule/process. In cases of severe SARS-CoV-2-infected patients, there is a markedly raised level of cytokines (a cytokine storm), and IFN-γ reflects a state of hyperinflammation, which may break down the blood–brain barrier (BBB) and thus expose the brain to all types of reactive/toxic agents. A cytokine storm is more easily triggered in patients suffering with diseases linked to chronic inflammation such as diabetes. The SARS-CoV-2 virus blocks melatonin from the pineal gland and thereby contributes to the cytokine storm and elevation of circulating lipopolysaccharides (LPS) [166]. The LPS can induce cellular lipid peroxidation and induce oxidative stress and cellular deleterious events. Therefore, the cytokine release in PD may be related to melatonin.

Interestingly, there are other parallel associations between SARS-CoV-2 and parkinsonism [167]. These are based on a number of common clinical and pathological features shared including hyposmia, pathology in the basal ganglia of one SARS-CoV-2 infected patient [168], and the activation of microglia mediated via pro-inflammatory cytokines (such as, IL-1β, TNF-α) release, which may then increase the risk for PD and the potential of protein aggregation due to disregulation of protein homeostasis [169]. Collectively, these finding clearly support the involvement of the α-synuclein-associated inflammatory mechanisms in neurodegeneration. However, at present, it is too early to predict if the SARS-CoV-2 infected patients will develop any parkinsonian motor features. Nevertheless, it may be noteworthy to monitor and follow up the acutely affected patients that exhibit continued neurological defects such as hyposmia and persistent confusion coupled with subsequent findings from post-mortem studies [170], particularly as the pathophysiology has been reported to produce changes in the dopamine synthesis pathways [171]. This may be related to the reduction of angiotensin converting enzyme 2 receptor on dopamine neurons in PD, since the dopamine synthesis enzyme dopamine decarboxylase co-expresses with angiotensin converting enzyme 2 [172,173]. Subsequently, a reduction of dopamine over a period of time may produce parkinsonian features or motor deficits.

### 3.5. Neuroinflammation

Based on post-mortem studies, α-synuclein pathology in PD probably originates in the periphery, starting from the gut and/or the olfactory system. It has been suggested that the translocation of bacteria (or infectious agents) and their products coupled with enhanced intestinal permeability may provide a conducive environment for α-synuclein aggregation. Strikingly, short chain fatty acids and extracellular fibers produced from bacteria resident in the gut have been associated to α-synuclein modification and accumulation [174]. In early PD, it then spreads from the gut via the vagus nerve and/or the olfactory system to brain regions. Subsequently as the disease progresses, it further spreads and accumulates in the SN pars compacta of the mid-brain and other brain regions [175,176]. It spreads within the neurons in a prion-like fashion. However, the Braak’s “bottom–up hypothesis” has been challenged [106], since only in about 50% PD patients can it be attributed to gastro-intestinal triggering of the disease. Recently, the “top–down hypothesis” has been postulated, suggesting that the disease process starts due to cortical–striatal excitatory stress, leading to synaptic dysfunction and followed by a striatonigral retrograde process, including α-synuclein pathology [6,177]. Furthermore, the “threshold hypothesis” suggested by Engelender and Isacson [178] collectively considers various vulnerability sources including lysosomal clearing functions, different anatomical structures, cell types, and genetic factors [85,179]. The characteristic manifestation of asymmetric degeneration in PD [180] may be better explained by the “threshold hypothesis” in contrast to the “bottom–up hypothesis’’ [6]. The build-up of α-synuclein aggregates may prompt microgliosis and recruit CD4+ and CD8+ T cells. CD4+ cells play a pivotal role in adaptive immune responses. α-Synuclein assigned CD4+ and CD8+ T cells in PD recognize proteins attached to MHC II on the microglia. The elements prompting the onset of the peripheral α-synuclein pathology (Figure 1) are still not clearly defined. However, it appears that it evokes peripheral inflammation. This contention is supported by in vitro studies exhibiting responsive circulating T cells from PD patients to modified α-synuclein. Subsequently, a sustained state of peripheral inflammation may damage the blood–brain barrier (BBB). The BBB strictly regulates the flow of immune molecules to the brain and rapidly destroys immune cells by apoptotic mechanisms, since the neurons are susceptible to immune attack. Therefore, a “leaky” BBB can literally open the flood gates and expose the brain to an array of peripheral inflammatory molecules, lymphocytes, and potential endo/exotoxins, which may contribute to the pathogenesis of the disorder (Figure 1). Indeed, in vivo studies using histological markers have demonstrated an enhanced permeability of the BBB in the striatal areas of PD patients [181]. This concords with the multifactorial hypothesis proposed in the etiology of the malady. Thus, modified α-synuclein inclusions somehow temper/prime the CD 4+ and CD8+ T cells of the peripheral adaptive immune system, which then deteriorate the BBB and may exacerbate the destruction of vulnerable dopamine neurons in the SN. Regulatory T cells (Treg) control unwanted immune responses. In PD, Tregs are reported to be impaired in blocking effector T-cell proliferation in vitro [182]. Furthermore, the Tregs are unable to quell the pro-inflammatory cytokine release from the T- effector cells [183]. These findings are suggestive of a threatening role of inflammation perhaps in the late phase of the illness.

Therefore, infectious agents (such as virus) that activate the onset of inflammation possess the potential to enhance the cellular destruction (Figure 1). Alternatively, a dysfunctional immune system could also generate similar deleterious effects. There could be a genetic component responsible for the malfunctioning inflammatory responses, such as PD genes LRRK2 and Parkin, which can affect the immune reactions [184]. 

The normal physiological response to viral infection is the activation of the hosts immune system as a defense mechanism to eradicate the foreign organism. However, in the diseased state, many factors come into play. For instance, in the case of PD, a leaky BBB may allow the entry of neurotoxic pro-inflammatory cytokines as the invading agent into the brain. PD is an age-related disorder, and the elderly have a compromised immune system. This may account for the increased risk of PD with advancing age. In addition, increasing age is related to enhanced permeability of BBB [185] and may partly contribute to the leaky BBB.

## 4. Conclusions

The significance of the modified α-synuclein aggregates in the pathogenesis of PD is demonstrated by its appearance in close proximity to dying/dead nigral dopaminergic neurons in the early asymptomatic phase of the disease. It appears to serve the prime purpose as a cellular executioner and triggers a rampant of neuronal destruction in the SN and other areas.

Therefore, in order to execute cellular warfare, α-synuclein:

Summons “rebels” (reactive molecules or a compound interacting with toxins/infectious agents) and initiates cytotoxic processes such as:

- ROS-mediated OS
- inflammation via pro-inflammatory cytokines (IL-1β, TNF-α and others)
- microgliosis

Furthermore, it causes cellular dysfunctions:

- iron dyshomeostasis by operating as ferrireductase
- ferroptosis
- release of dopamine from vesicles, thus augmenting dopamine metabolism resulting in OS
- mitochondrial dysfunction
- immunological alterations

From all the available evidence, we conclude that sporadic PD is based on different triggers with a variety of mechanisms to release the disorder: (1) multiple genetic alterations with low penetrance, (2) triggers causing either rapid onset of PD, such as MPTP, which disturbs respiratory activity and interacts with α-synuclein but does not show LB formation (3), triggers such as influenza A viral infection, which causes postencephalitic parkinsonism only years after viral infection and is without LB formation. Experimental work demonstrates that the dose and duration of neurotoxic influence may be decisive to explain not only time of onset and degree of disease severity but also the preparedness and facilitation to LB formation. As such, alterations underlying α-synuclein pathology are regarded as the most dangerous mechanisms to release PD and to build up LB, which per se add toxicity to destroy NM-containing catecholaminergic neurons.

Neuroinflammation appears to represent an epiphenomenon to the misfolded α-synuclein inclusions; nevertheless, it skillfully orchestrates processes that amplify neuronal destruction. This contention is supported by the findings obtained from virus-induced parkinsonism, thereby etching the immune system as a formidable contender in the labyrinth of neurodegenerative processes. Although there are disparities between the cellular and pathological changes and progression in PD and virus-related parkinsonism, this is probably due to the involvement of other aetiological factors. This concords with multifactorial hypotheses in PD. Indeed, it appears highly likely that there is an interplay between α-synuclein aggregates and genetic predisposition coupled with increasing age. Perhaps an early contact with an infectious agent or some neurotoxin that triggers the inflammatory mechanism may confer a susceptibility or facilitate neurodegeneration at a later age. Alternatively, some genetic abnormality may generate a dysregulation of a key protein, leading to α-synuclein accumulation and the related cellular deleterious events.

Therefore, even though the precise causal agents/molecules underlying the etiology of PD remain elusive, nevertheless, the experimental, clinical, and post-mortem findings are suggestive of putative cellular and molecular mechanism(s) that can be targeted in the management of the illness [186]. The first area of “strike” would be to employ molecules/compounds that stabilize misfolded α-synuclein so as to impede its aggregation or a chemical that disaggregates the oligomers. It may be highly effective to use drugs that increase the destruction and clearance of α-synuclein inclusions and increase regenerative processes. This would block its ability to summon other cytotoxic mechanisms.

## Data Availability

Not applicable.
